# PCR-Restriction Fragment Length Polymorphism analysis of *fljB* gene in *Salmonella enterica* subspecies *enterica* serovar Typhimurium isolated from avians

**Published:** 2010-12

**Authors:** M Dilmaghani, M Ahmadi, T Zahraei Salehi, A Talebi, R Darvishzadeh

**Affiliations:** 1 Department of Microbiology, Faculty of Veterinary Medicine, University of Urmia,Urmia, Iran.; 2 Department of Microbiology, Faculty of Veterinary Medicine, University of Tehran, Tehran, Iran.; 3 Department of Clinical Sciences, Faculty of Veterinary Medicine, University of Urmia,Urmia, Iran.; 4 Department of Agronomy and Plant breeding University of Urmia, Urmia, Iran.

**Keywords:** *Salmonella* Typhimurium, *fljB* gene, PCR-RFLP, Avians

## Abstract

**Background and Objectives:**

Economic constraint of diseases arising from *Salmonella* Typhimurium causes the study of this zoonotic organism more important. Most studies on identification and characterization of *S*. Typhimurium are conducted at DNA level. Flagellin genes (*fliC* and *fljB* genes encoding phase-1 and phase-2 flagella, respectively) are useful as a model system for studying genetic differentiation. The objectives of the present study were to identify the polymorphism of *fljB* among avians in different regions by the PCR-RFLP method.

**Materials and Methods:**

Fifty-two *S*. Typhimurium isolates out of 1,870 intestine samples were identified using culture and serotyping as well as multiplex-PCR (broiler (n=13), layer (n=12), duck (n=5), goose (n=5), sparrow (n=8), canary (n=3), pigeon (n=5) and casco parrot (n=1)). Amplification of *fljB* gene was performed and amplified products subjected to restriction digestion with H*ha* I enzyme.

**Results:**

Two RFLP patterns generated DNA fragments between approximately 50 to 800 bps. Pattern A was observed in 33 (63.46%) and pattern B in 19 (36.54%) of isolates. *Salmonella* Typhimurium recovered from 13 broilers (ten with pattern A and 3 with pattern B) and 8 sparrow (three with pattern A and 5 with pattern B) showed both A and B patterns. Twelve layers, 5 pigeons and 3 canaries showed pattern A and 5 ducks, 5 geese and one casco parrot showed pattern B. None of these patterns was allotted for a special region.

**Conclusion:**

The results of the present study showed that *fljB* gene is highly conserved among avians in different geographical regions, suggesting not only the importance of *fljB* gene in survival of organism in different environmental conditions but also the relation between proteins encoded by *fljB* gene and serotyping scheme.

## INTRODUCTION

Members of the genus *Salmonella* colonizes vertebrate hosts, with outcomes ranging from subclinical to systemic infection with high mortality.

Animal infection has direct economic consequences, but asymptomatic carriage, leading to direct or indirect transmission to humans, maybe even more important (1). Molecular methods have shown that the genus *Salmonella* consists of only two species: *S.enterica* and *S.bongori*. *Salmonella enterica* is divided into the following six subspecies: *S. enterica* subsp. *enterica*, *S. enterica* subsp. *salamae*, *S. enterica* subsp. *arizonae*, *S. enterica* subsp. *diarizonae*, *S. enterica* subsp. *houtenae* and *S. enterica* subsp. *Indica* (2). Members of *Salmonella enterica* subspecies *enterica* are mainly associated with warm-blooded vertebrates and are usually transmitted by ingestion of food or water contaminated feces. *Salmonella enterica* subspecies *enterica* serovar Typhimurium as a ubiquitous serovar is capable of causing systemic disease in human and a wide range of host animals and usually induce a self-limiting gasteroenteritis (3). This non-typhoidal *Salmonella* is a major cause of diarrheal disease in both industrialized and developing countries (4).

*S*. Typhimurium express two antigenically distinct flagellins encoded by the *fliC* and *fljB* genes. The alternative expression of these two genes is known as phase variation and it occurs on a time scale of the order of 103-105 generations (5). Flagellin genes are about 1,500 bp in length, with two conservative terminal regions and a highly variable central region. The corresponding domain of flagellin is located on the surface of the filament and constitutes the flagellar epitopes (6). The *fljBA* operon contains *hin*, encoding Hin recombinase; *fljB*, encoding phase-2 flagellin; *fljA*, encoding a repressor for *fliC* gene. The Hin recombinase catalyzes the reversible inversion of a 993-bp segment of the chromosome containing a promoter. This sequence has two 14- bp inverse boundary repeats. In one orientation, the promoter directs transcription of the *fljB* and *fljA* genes, inducing repression of the *fliC* gene. In the other hand, *fljB* and *fljA* are not expressed and consequently *fliC* is switched on and phase-1 flagellin expressed. This mechanism is thought to play an important role in adaptation of *Salmonella* to warm-blooded animals. The biological significance of phase variation in *S*. Typhimurium is most likely to provide defense against antibodies of the host organism (7, 8).

Flagellin genes are useful as a model system for studying genetic differentiation because they are so flexible to various mutations that their mutation products are functional unless the 5′ and 3′ coding frames, which encode the regions important for secretion and polymerization of flagellin, are changed (9).

In the present study, identification of *S*. Typhimurium isolates were performed with classical culture technique and serotyping as well as multiplex-PCR. The objective was to identify the polymorphism of *fljB* gene among avians in different regions of Iran using the PCR-RFLP method.

## MATERIALS AND METHODS

**Collection of Samples**. In the time span between June 2009 to June 2010, a total of 1, 870 samples from intestinal contents of avians (broiler, layer, sparrow, duck, goose, pigeon, canary and casco parrot) were collected from different geographical regions including North and Northwest of Iran. All samples were taken to the microbiology laboratory of Urmia Veterinary Faculty on ice to be refrigerated and processed in the same day and in sterile conditions. Each sample (1-2 gram) was enriched in Selenit broth tubes and incubated overnight at 37°C. A loop of each tube was streaked onto CHROMagar™ Salmonella medium (CHROMagar Microbiology, Paris, France) plates incubated at 37°C for 24 h. Suspected colonies (typical mauve colonies in CHROMagar™ Salmonella medium (CAS)) were subcultured on Salmonella-Shigella agar medium (Merk, Germany) plates to obtain pure cultures. All pure isolates were Gram-stained and tested for their characteristic biochemical reactions.

The bacteria were maintained on MacConkey agar plates. For longer storage, isolates grown in Leuria- Bertani broth were mixed with glycerol to obtain final concentration of 15% glycerol and kept at −80°C (10).

**Serotyping of isolates**. All isolates presumptively identified as *Salmonella*, were serotyped at Razi vaccine and serum research institute (Iran) using slide agglutination test, and *S*. Typhimurium isolates were identified according to their serotyping formula: 1, 4,5, 12: i: 1, 2. Finally, a total of 52 *S*. Typhimurium were identified from various avians; broiler (n=13), layer (n=12), duck (n=5), goose (n=5), sparrow (n=8), canary (n=3), pigeon (n=5) and casco parrot (n= 1). To confirm the results of serotyping, all isolates were tested by multiplex-PCR.

**Genomic DNA extraction**. DNA extraction of all *Salmonella* isolates were performed from overnight culture in buffered peptone water by Genomic DNA purification kit (Fermentas, Germany) with some modifications including the use of phenol-chloroform- isoamylalcohol (25: 24: 1) instead of chloroform which was indicated in DNA extraction protocol. Purity of DNA was tested spectrophotometrically at wave length of 260 and 280 nm (Eppendorf biophotometer plus 6132, Germany). Extracted DNA was diluted to give a final concentration of 50 ng.

**Multiplex-PCR reaction for isolates**. All isolates tested by multiplex-PCR have been described previously (11) with some modifications (12). Four sets of primers used in this study (Table 1), include: Rfbj, FljB, FliC and ST-139 and ST-141 which their target genes encoding O4 antigen (663bp), H2:1, 2 (526bp), H1: i (183bp) and InvA (284bp) respectively. In the present study, *S*. Typhimurium ATCC 1730 was used as positive control. Multiplex-PCR was performed in a final volume of 25 µl containing: PCR buffer (10mM Tris-HCl, 50 mM KCl, 1.5 mM MgCl, ph 8.7), dNTP (200 µM), primer (1 µM) and Smartaq™ DNA polymerase, (1U) (Cinnagen, Iran) and template DNA (50 ng).

**Table 1:** Primers characteristics used in this study.

Primer	Primer	Primer length (bp)	Sequence	Amplified fragment size (bp)	Reference
Flic-s	*fliC*	24	3′- CCCCCTTGACCATTCTACCGATA	183	Lim et al. (2003)
Flic-as		24	3′- CCGTATAGGACATTGTCAACGTCG		
ST-139	*invA*	26	3′- AACGGGCTTGCACCGCTATTAAAGTG	284	Rhan et al. (1992)
ST-141		22	3′- CCAAGGAAACTGCCACGCTACT		
FljB-s	*fljB*	24	3′- CCAATGTCTTCGGCATGGTAAGCA	526	Lim et al. (2003)
Flj-as		24	3′- GGCTTCAGCAATGATAGCTGCCAT		
Rfbj-s	*rfbJ*	24	3′- CATAGTTCAACCTTGACCACGACC	663	Lim et al. (2003)
Rfbj-as		24	3′- ACGAATGGTTATTTCGGCCTTCGG		

For negative control, sterile water was added instead of nucleic acid. PCR reaction was performed in a DNA thermocycler (Model CP2-003, Corbett, Australia) as follows:

an initial denaturation at 95°C for 5 min., 35 cycles of denaturation at 95°C for 1min, annealing at 65°C for 1min, elongation at 72°C for 30s and final 7min extension period at 72°C. Amplified products were separated by 2% agarose gel electrophoresis at 80 V for 1 h and photographed under UV illuminator.

### PCR-RFLP of *S*. Tyhpimurium isolates

**Amplification of** ***fljB*** **gene**. Amplification of *fljB* gene was performed by primer pair previously reported and used to amplify a fragment of approximately 1.5 kb (13). The sequences of primers were as follows:

FSa2, CAAGTAATCAACACTAACAGTC

rFSa2, TTAACGTAACAGAGACAGCAC.

The PCR reaction was carried out in a volume of 50 µl containing: PCR buffer (50 mM Tris-HCl, 50 mM

KCl, 2.5 mM MgCl, pH 8.7), dNTP (400 µM), each primer (1 µM) and Smartaq™ DNA polymerase (2U) (Cinnagen, Iran) and 50 ng of extracted DNA. For negative control, sterile water was added instead of nucleic acid. Amplification was performed in a DNA thermocycler (Model CP2-003, Corbett, Australia) as follows:

an initial denaturation at 95°C for 3 min, followed by 45 cycles of 1 min. at 94°C, 1 min at 58°C and 1 min at 72°C with a final step at 72°C for 5 min. The amplified products were visualized by gel electrophoresis using 10 µl of final reaction mixture on 0.8% agarose gel for 1 h at 100 V.

**Restriction digestion of PCR-amplified** ***fljB*** **gene**. 10–15 µl of each PCR-amplified product was digested with 10 U of H*ha* I restriction enzyme at 37°C for 3 h according to manufacturer‘s instructions (Fermentas, Germany). Digested PCR products were separated by electrophoresis at 80V on 2% agarose gel for about 2h and photographed under UV illuminator.

## RESULTS

**Fig. 1:**
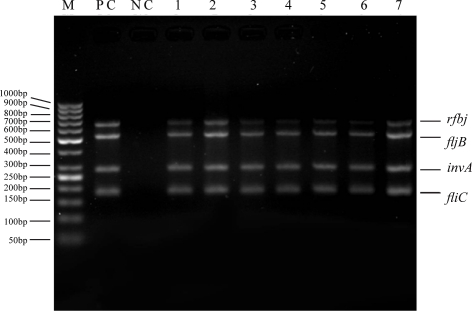
The results of multiplex-PCR assay. Lane M: 50bp DNA Ladder (Fermentas, Germany); Lane PC: Positive control; Lane NC: Negative control; Lane 1 to 7: *S*. Typhimurium isolates.

**Identification of** ***S**.* **Tyhpimurium**. Out of 1, 870 samples, 52 were isolated as *S*. Tyhpimurium (broiler (n=13), layer (n=12), duck (n=5), goose (n=5), sparrow (n=8), canary (n=3), pigeon (n=5) and casco parrot (n=1)). The results of serotyping were confirmed with multiplex-PCR. Primers listed in table 1 for multiplex-PCR could successfully amplify the expected sizes: 183, 284, 526 and 663 bp from *fliC*, *invA*, *fljB* and *rfbJ* genes, respectively (Fig. 1). From negative control, no PCR product was obtained.

**PCR amplification of** ***fljB*** **gene**. In all 52 *S*. Typhimurium isolates, a 1.5 kb fragment from *fljB* gene was amplified and no variation in gene size was detected on gel electrophoresis.

**Fig. 2:**
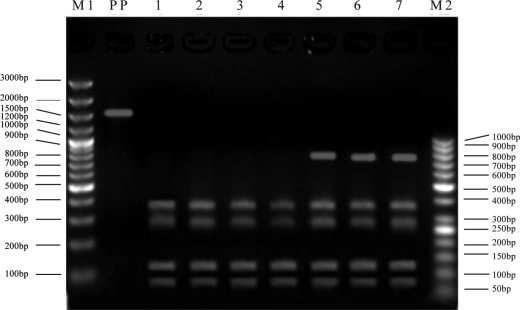
PCR and RFLP profiles of *fljB* gene after digestion with H*ha* I. Lane M1: 100bp plus DNA Ladder (Fermentas, Germany); Lane PP: PCR product before digestion; Lane 1-4: Profile A; Lane 5-7: Profile B; Lane M2: 50bp DNA Ladder (Fermentas, Germany).

**Restriction fragment length polymorphism of** ***fljB*** **gene**. PCR-RFLP analysis of *fljB* gene, using H*ha* I restriction enzyme, showed 2 distinct groups: A and B (Fig. 2) are generated DNA fragments between 50bp to 800 bp in size. The RFLP pattern A was observed in 33 (63.46%) and pattern B in 19 (36.54%) of isolates.

*S*. Typhimurium isolates recovered from 13 broilers (ten of which pattern A and 3 pattern B) and 8 sparrows (three of which pattern A and 5 pattern B) showed both A and B patterns, but 12 layers, 5 pigeons and 3 canaries showed pattern A and 5 ducks, 5 geese and one casco parrot showed pattern B (Table 2). In all geographical regions of this study all RFLP patterns (A and B) were observed and no pattern was allotted for a special region.

**Table 2:** Distributions of RFLP profiles among different host species.

Avians	No. of isolates	RFLP profiles	
		
		A	B
Broiler	13	10	3
Layer	12	12	--
Sparrow	8	3	5
Duck	5	--	5
Goose	5	--	5
Pigeon	5	5	--
Canary	3	3	--
Casco parrot	1	--	1

Total	52	33	19

## DISCUSSION

In *Salmonella*, flagella are a 55 kDa monomeric protein encoded by *fliC* and *fljB* that assemble to form the filament structure of the flagellar apparatus necessary for bacterial motility (14). Flagella (H antigen) on the surface of *S*. Typhimurium have been characterized as virulence factor that help the bacteria move toward and adhere to host cells (15). The amino and carboxyl termini of flagellin are quite conserved, not only between *fliC* and *fljB*, but also in flagellin from different serotypes. In contrast, the central portion of the protein is hyper variable and contains most of the antigenic residues (16, 17). Antigenic polymorphism of flagella seems to have been generated by the accumulation of ordinary genetic events in flagellin genes, such as point mutations, deletion and insertions (9).

Isolation and identification of *S*. Typhimurium with CAS medium and biochemical tests followed by serotyping and multiplex-PCR of isolates were performed in present study. There were no differences in sizes of PCR-amplified *fljB* gene on gel electrophoresis. These results agree with other studies (13, 18–20) and other researchers which used *fljB* gene as a marker for identification of serovars in company with other genes since there is just one copy of this gene in *Salmonella* containing phase-2 flagellin (17, 21–23).

It is well known that *fljB* locus show extensive heterogeneity among different *Salmonella* strains. This is due to integration of different prophage at the tmRNA gene adjacent to the *fljB* locus (24).

Although located in chromosome structure, *fljB* may not be detected serologically in some isolates. According to the results of serotyping and multiplex-PCR, this phenomenon was not observed in present study.

Analysis of the *flj* operon of *Salmonella* 9, 12,:l,v:- indicated that loss of phase 2 flagellar antigen expression occurred through deletion of the *hin* gene and adjacent DNA, thereby blocking the phase 2 flagellar gene in the off position (18). Sequences specific for *S*. Typhimurium and phage type DT 104 and U302 were present in *Salmonella* serovar 4, 5, 12:i:-, suggesting that it is a monophasic *S*. Typhimurium variant (25). Leader et al**. (2009) showed that an amplicon of *fljB* was observed for 100% of *S*. Typhimurium isolates, while 98% (47 of 48 isolates) of *Salmonella* serovar 4 [5] 12: i:- isolates produced no amplification. The results of serotyping and biochemical tests in their study could indicate that the isolate has a different mutation leading to the loss of the second-phase flagellar antigen (26). In another study on *Salmonella enterica* serovar 4, [5], 12:i:-, 94 of 116 isolates were PCR-negative for all variants of the *fljB* gene coding for the phase-2 flagellar antigen. Hopkins et al**. (2010) concluded that monophasic strains in which the phase-2 flagellar antigen is not detected serologically but can be detected by PCR may contain deletions in a part of *fljB* or *fljB* promoter controlling expression may become locked in one position that leave the H: 1, 2-specific PCR primer binding sites intact, or they may represent ‘serotype inconsistent’ strains (27). Other studies showed that mutations in steps leading to the formation of the basal body-hook structure do not express the flagellar filament structural genes, *fliC* and *fljB*, due to negative regulation by FlgM (28). Haung et al. (2007) showed that some regulators and factors including SirA, BarA, and RcsC/B influence expression of flagellar genes by regulating FlhDC, which is the global regulator of flagellar and motility related chemotaxis genes in *Salmonella* and *E. coli*. The expression of flagella-related genes is also affected by environmental factors, such as osmotic or acid stress. (29).

H*ha* I restriction enzyme which was previously used (13) for generation of RFLP pattern for *fljB* gene could digest all PCR products of present study yielding 2 RFLP patterns. This finding is an agreement with previous study by Dauga et al. (1998) in 11 *S*. Typhimurium isolates corresponding to six phage types, but not in agreement with Jong et al. (2010) study which could be due to insufficient number of *S*. Typhimurium isolates (n=2). Dauga et al. (1998) demonstrated that endonuclease HphI showed fewer profiles than H*ha* I and fewer antigen or serovar-specific patterns than H*ha* I.

Genotypic diversity in two flagellin genes, *fliC* and *fljB*, encoding phase-1 and phase-2 flagellin of *Salmonella enterica*, offers a potential biomarker for *Salmonella* subtyping (20). In one study (18), the profiles of the insertion element *IS*200, which has been shown to provide phylogenetic markers for serogroup D1 *Salmonella*, were analyzed in relation to the restriction fragment length polymorphisms of the phase 2 flagellar gene. Together they provide unequivocal evidence that *Salmonella* 9, 12:l, v:2 arose from a strain of *S*. Goettingen. The closely related antigens in *S*. Typhimurium and *S*. Mendoza gave similar profiles. The RFLP analysis of *fljB* with A*lu*I showed that serological reactivity always precisely coincided with restriction ftagment polymorphism. This was also indicated in previous studies (30).

In the present study, the size of DNA in RFLP profile varied between 50bp to 800bp in profile B and in profile A was between 50 and 400bp. The variation of *fljB* gene RFLP profiles, as it was mentioned before, might be due to different host species, genetic alteration in restriction sites and some point mutations which may be observed by sequencing or PCR-SSCP method. Pattern A was predominant (63.46%) and pattern A and pattern B were observed in both broilers and sparrows which showed close relation of flagellin genes in these host species. Sparrows nest in regions close to poultry farms and this may prepare the way for transmission of *S*. Typhimurium between these hosts. Ducks, geese and casco parrot together with pigeons and canaries showed identical RFLP patterns B and A, respectively. The plausible explanation for this may be due to the condition in which ducks and geese are bred closely, but it needs more isolates to compare, especially for casco parrot. We cannot allocate each unique pattern for a special host (For example; Pattern A for pigeons and canaries) and these patterns cannot show *fljB* gene characteristics in special host species because more isolates need to be tested.

In conclusion, the structure of *fljB* gene in *S*. Typhimurium is highly conserved among different host species. This means that conserved region of this gene is vital for *S*. Typhimurium to protect it in different conditions, as it was mentioned before. The findings of present study in agreement with previous studies confirmed that flagellin genes (*fljB* gene in present study) encode proteins on *Salmonella* surface in relation to serotyping scheme. These conserved parts might be good candidates to develop vaccine against *S*. Typhimurium and other *Salmonella* which harbor the same conserved regions. This will be achieved through further studies on sequences of flagellin genes.
